# 

**Published:** 1900-06-30

**Authors:** 


					THE HOSPITAL. ''The standard of highest purity."?Lancet. June 30th, 1900.
CADBURY'S ??*. U ** CADBURY'S
COOOA
COCOA
NO DRUGS OR ALKALIES USED.
No. 718) Vol. XXVIII. [?S??3?1 Saturday,
June 30, 1900.
?Sub^ription Terns.] pRICE TWOPENCE.

				

